# 2,9-Dimethyl-1,10-phenanthrolin-1-ium (6-carb­oxy-4-hy­droxy­pyridine-2-carboxyl­ato-κ^3^ *O* ^2^,*N*,*O* ^6^)(4-hy­droxy­pyridine-2,6-dicarboxyl­ato-κ^3^ *O* ^2^,*N*,*O* ^6^)zincate(II) 2.35-hydrate: a proton-transfer compound

**DOI:** 10.1107/S1600536811052445

**Published:** 2011-12-10

**Authors:** Zohreh Derikvand, Helen Stoeckli-Evans, Andya Nemati

**Affiliations:** a Faculty of Science, Department of Chemistry, Khorramabad Branch, Islamic Azad University, Khorramabad, Iran; b Institute of Physics, University of Neuchâtel, Rue Emile-Argand 11, CH-2000 Neuchâtel, Switzerland; c Iran Compiling Encyclopedia Foundation, Tajrish, Tehran, Iran

## Abstract

In the title compound, (C_14_H_13_N_2_)[Zn(C_7_H_3_NO_5_)(C_7_H_4_NO_5_)]·2.35H_2_O, the Zn^II^ atom is coordinated by two N atoms and four O atoms from the carboxyl­ate groups of the 4-hy­droxy­pyridine-2,6-dicarboxyl­ate and 6-carb­oxy-4-hy­droxy­pyridine-2-carboxyl­ate ligands, forming a distored octa­hedral geometry. In the anion, the two pyridine rings are inclined to one another by 87.75 (13)°. Two types of robust O—H⋯O hydrogen bond synthons, *viz. R*_2_^2^(16) and *R*_6_^6^(42), link the anions to form a two-dimensional network parallel to the *bc* plane. Furthermore, O—H⋯O, N—H⋯O, N—H⋯N and weak C—H⋯O hydrogen bonds connect the two dimensional networks, forming a three-dimensional structure. In the crystal, there are also C—H⋯π and π–π inter­actions [centroid–centroid distances of 3.5554 (18) and 3.7681 (18) Å], and C=O⋯π inter­actions [O⋯centroid distance = 3.117 (2) Å] present. One of the three crystal water molecules shows an occupancy of 0.35.

## Related literature

For related structures, see: Aghabozorg *et al.* (2007*a* ▶,*b* ▶,*c* ▶, 2008*a* ▶,*b* ▶,*c* ▶); Derakhshandeh *et al.* (2010 ▶); Moghimi *et al.* (2005*a* ▶,*b* ▶).
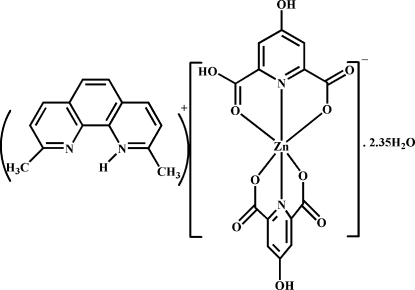


## Experimental

### 

#### Crystal data

(C_14_H_13_N_2_)[Zn(C_7_H_3_NO_5_)(C_7_H_4_NO_5_)]·2.35H_2_O
*M* *_r_* = 680.19
Monoclinic, 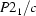

*a* = 11.0687 (18) Å
*b* = 9.7888 (14) Å
*c* = 25.776 (4) Å
β = 94.160 (19)°
*V* = 2785.4 (7) Å^3^
*Z* = 4
Mo *K*α radiation
μ = 0.96 mm^−1^
*T* = 223 K
0.38 × 0.15 × 0.15 mm

#### Data collection

Stoe IPDS diffractometer
Absorption correction: multi-scan (*MULscanABS* in *PLATON*; Spek, 2009 ▶) *T*_min_ = 0.972, *T*_max_ = 1.000
20517 measured reflections
5464 independent reflections
3242 reflections with *I* > 2σ(*I*)
*R*_int_ = 0.079

#### Refinement

*R*[*F* ^2^ > 2σ(*F* ^2^)] = 0.039
*wR*(*F* ^2^) = 0.081
*S* = 0.82
5464 reflections
437 parameters
12 restraints
H atoms treated by a mixture of independent and constrained refinement
Δρ_max_ = 0.39 e Å^−3^
Δρ_min_ = −0.82 e Å^−3^

### 

Data collection: *EXPOSE* in *IPDS-I* (Stoe & Cie, 2000 ▶); cell refinement: *CELL* in *IPDS-I*; data reduction: *INTEGRATE* in *IPDS-I*; program(s) used to solve structure: *SHELXS97* (Sheldrick, 2008 ▶); program(s) used to refine structure: *SHELXL97* (Sheldrick, 2008 ▶); molecular graphics: *PLATON* (Spek, 2009 ▶) and *Mercury* (Macrae *et al.*, 2008 ▶); software used to prepare material for publication: *SHELXL97*, *PLATON* and *publCIF* (Westrip, 2010 ▶).

## Supplementary Material

Crystal structure: contains datablock(s) I, global. DOI: 10.1107/S1600536811052445/lh5386sup1.cif

Structure factors: contains datablock(s) I. DOI: 10.1107/S1600536811052445/lh5386Isup2.hkl

Additional supplementary materials:  crystallographic information; 3D view; checkCIF report

## Figures and Tables

**Table 1:** Hydrogen-bond geometry (Å, °) *Cg*1 is the centroid of the N1,C1–C5 ring.

*D*—H⋯*A*	*D*—H	H⋯*A*	*D*⋯*A*	*D*—H⋯*A*
N3—H3⋯N4	0.87	2.37	2.721 (3)	105
N3—H3⋯O1*W*	0.87	2.01	2.848 (4)	161
O5—H5⋯O2*W*	0.83	1.76	2.570 (4)	166
O7—H7⋯O2^i^	0.83	1.66	2.402 (3)	147
O10—H10⋯O3^ii^	0.83	1.75	2.562 (3)	166
O1*W*—H1*WA*⋯O9^iii^	0.83 (3)	2.02 (4)	2.833 (4)	169 (4)
O1*W*—H1*WB*⋯O4^iv^	0.84 (4)	2.18 (4)	2.960 (4)	156 (3)
O2*W*—H2*WA*⋯O8^v^	0.82 (4)	1.98 (4)	2.738 (4)	155 (4)
O2*W*—H2*WB*⋯O4^vi^	0.81 (2)	2.25 (3)	3.046 (4)	171 (4)
O3*W*—H3*WA*⋯O4	0.83 (2)	1.75 (5)	2.530 (7)	157 (13)
O3*W*—H3*WB*⋯O2*W*^v^	0.82 (2)	2.21 (4)	2.750 (9)	123 (3)
C27—H27*C*⋯O3^vii^	0.97	2.58	3.504 (4)	160
C22—H22⋯*Cg*1^viii^	0.94	2.76	3.634 (4)	155

Symmetry codes: (i) 
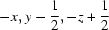
; (ii) 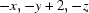; (iii) 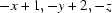; (iv) 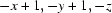; (v) 
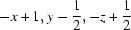
; (vi) 
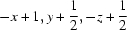
; (vii) 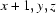; (viii) 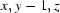.

**Fig. 1.**
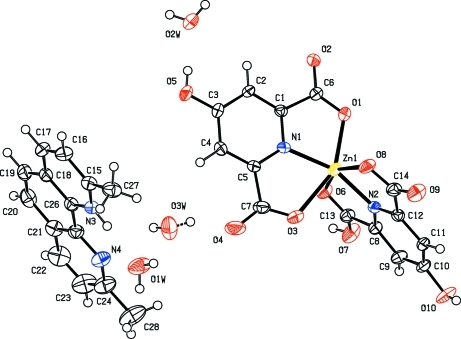
The molecular structure of the title compound, showing the displacement ellipsoids at the 50% probability level.

**Fig. 2.**
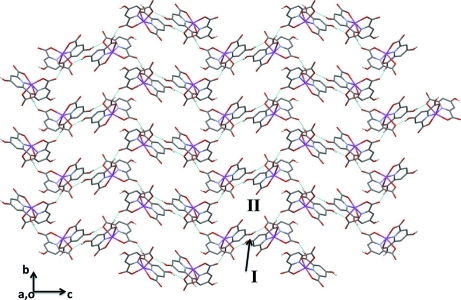
A view along the a axis of the robust O–H···O hydrogen bond synthons, (I) R22(16) and (II) R66(42) forming a two-dimensional network parallel to the bc plane [the dashed cyan lines show donor-acceptor distances of hydrogen bonds].

**Fig. 3.**
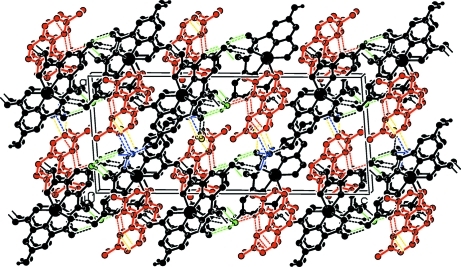
Crystal packing of the title compound viewed along the b axis. The O—H···O and N—H···N,O hydrogen bonds are shown as dashed lines [anion is black; cation is red; water molecules are blue, green and yellow].

**Fig. 4.**
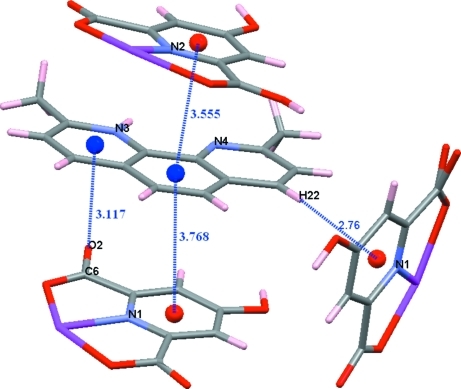
A view of the π···π stacking interactions involving the aromatic rings of the anions [(hpydc)2- and (hpydcH)-] and the cations [(dmpH)+], and the C═O···π and C–H···π interactions (see Comment section and Table 1 for details).

## supplementary crystallographic information

### Comment

The crystal structure of some proton transfer complexes where 4-hydroxypyridine-2,6-dicarboxlic acid (hypydcH_2_) is the proton donor have been reported on previously (Derakhshandeh *et al.*, 2010; Aghabozorg *et al.*, 2007*a*, 2007*b*, 2007*c*, 2008*a*, 2008*b*, 2008*c;* Moghimi *et al.*, 2005*a*, 2005*b*). Herein, we report on the crystal structure of the title compound, obtained by the reaction of zinc(II)nitrate, with the same proton donor (hpydcH_2_) and the proton acceptor 2,9-dimethyl-1,10-phenanthroline (dmp).

The title compound contains of one [Zn(hpydc)(hpydcH)]^-^ anion, one (dmpH)^+^ cation and 2.35 uncoordinated water molecules (Fig. 1). A carboxylic acid proton has been transferred to an N atom of 2,9-dimethyl-1,10-phenanthroline. In the anions, the Zn^II^ atom is six-coordinated by two N atoms (N1 and N2) that occupy the axial positions, and four O atoms (O1, O3, O6 and O8) from the carboxylate groups of the (hypydc)^2–^ and (pydcH)^–^ ligands in the equitorial plane, so forming a distorted octahedral geometry. The (hypydc)^2-^ and (pydcH)^-^ ligands are almost perpendicular to one another, with a dihedral angle of 87.75 (13) ° between the two pyridine rings, (N1,C1–C5) and (N2,C8–C12). There is a short N—H···N interaction in the cation (Table 1).

In the crystal, the anions are linked *via* two types of robust O—H···O hydrogen bond synthons, type (I) *R*^2^_2_(16) and (II) *R*^6^_6_(42), forming a two-dimensional network lieing parallel to the *bc* plane (Fig. 2). Intermolecular O—H···O, N—H···O, N—H···N and weak C—H···O hydrogen bonds connect these two dimensional networks to form a three-dimensional arrangement (Table 1 and Fig. 3).

Another aspect of the crystal structure, illustrated in Fig. 4, is the presence of π–π interactions involving the pyridine rings, (*Cg*1 = N1,C1—C5) and (*Cg*2 = N2,C8—C12) of the anion and the central ring (*Cg*3 = C18—C26) of the phenantholinium cation: centroid-centroid distances are 3.7681 (18) Å for *Cg*1···*Cg*3^i^ [symmetry code: (i) *x* + 1, -*y* + 1/2, *z* + 1/2] and 3.5554 (18) Å for *Cg*2···*Cg*3^ii^ [symmetry code: (ii) *x* - 1, *y* + 1, *z*]. There is also a C—H···π interaction (C22—H22···*Cg*1^viii^, see Table 1) and a C═O···π interaction present [C6═O2···*Cg*4^iii^ = 3.117 (2) Å; symmetry code: (iii) *x* - 1, *y* + 1/2, -*z* + 1/2; *Cg*4 is the centroid of ring (N3,C15—C18,C26)].

The crystal structure of the title compound is isostructural to that of the nickel(II) complex, (dmpH)[Ni(hpydc)(hpydcH)]^.^2.35 H_2_O (Derakhshandeh *et al.*, 2010).

### Experimental

The reaction between 4-hydroxypyridine-2,6-dicarboxylic acid (100 mg, 1 mmol) in 10 ml water, 2,9-dimethyl-1,10-phenanthroline(dmp) (110 mg, 1 mmol) in 20 ml water and zinc(II)nitrate hexahydrate (90 mg, 0.5 mmol) in 5 ml water, in a 2:2:1 molar ratio, gave colourless crystals after slow evaporation of the solvent at room temperature.

### Refinement

The water H-atoms could all be located in difference Fourier maps. They were refined with distance restraints, O—H = 0.84 (2) Å and H···H = 1.35 (2) Å, with *U*_iso_(H) = 1.5*U*_eq_(O). The NH, OH and C-bound H-atoms were included in calculated postions and treated as riding atoms: O—H = 0.83 Å, N—H = 0.87 Å, C—H = 0.94 and 0.97 Å for CH and CH_3_ H atoms, respectively, with *U*_iso_(H) = k × *U*_eq_(O,*N*,*C*), where k = 1.5 for OH and CH_3_ H atoms and k = 1.2 for all other H atoms.

### Crystal data

**Table:**

(C_14_H_13_N_2_)[Zn(C_7_H_3_NO_5_)(C_7_H_4_NO_5_)]·2.35H_2_O	*F*(000) = 1398
*M**_r_* = 680.19	*D*_x_ = 1.622 Mg m^−^^3^
Monoclinic, *P*2_1_/*c*	Mo *K*α radiation, λ = 0.71073 Å
Hall symbol: -P 2ybc	Cell parameters from 8000 reflections
*a* = 11.0687 (18) Å	θ = 2.2–26.0°
*b* = 9.7888 (14) Å	µ = 0.96 mm^−^^1^
*c* = 25.776 (4) Å	*T* = 223 K
β = 94.160 (19)°	Rod, colourless
*V* = 2785.4 (7) Å^3^	0.38 × 0.15 × 0.15 mm
*Z* = 4	

### Data collection

**Table:**

Stoe IPDS diffractometer	5464 independent reflections
Radiation source: fine-focus sealed tube	3242 reflections with *I* > 2σ(*I*)
graphite	*R*_int_ = 0.079
phi rotation scans	θ_max_ = 26.1°, θ_min_ = 2.2°
Absorption correction: multi-scan (MULscanABS in *PLATON*; Spek, 2009)	*h* = −13→13
*T*_min_ = 0.972, *T*_max_ = 1.000	*k* = −12→12
20517 measured reflections	*l* = −31→31

### Refinement

**Table:**

Refinement on *F*^2^	Secondary atom site location: difference Fourier map
Least-squares matrix: full	Hydrogen site location: inferred from neighbouring sites
*R*[*F*^2^ > 2σ(*F*^2^)] = 0.039	H atoms treated by a mixture of independent and constrained refinement
*wR*(*F*^2^) = 0.081	*w* = 1/[σ^2^(*F*_o_^2^) + (0.0381*P*)^2^] where *P* = (*F*_o_^2^ + 2*F*_c_^2^)/3
*S* = 0.82	(Δ/σ)_max_ = 0.002
5464 reflections	Δρ_max_ = 0.39 e Å^−^^3^
437 parameters	Δρ_min_ = −0.82 e Å^−^^3^
12 restraints	Extinction correction: *SHELXL97* (Sheldrick, 2008), Fc^*^=kFc[1+0.001xFc^2^λ^3^/sin(2θ)]^-1/4^
Primary atom site location: structure-invariant direct methods	Extinction coefficient: 0.00093 (19)

### Special details

**Table:**

Geometry. Bond distances, angles *etc*. have been calculated using the rounded fractional coordinates. All su's are estimated from the variances of the (full) variance-covariance matrix. The cell e.s.d.'s are taken into account in the estimation of distances, angles and torsion angles
Refinement. Refinement of *F*^2^ against ALL reflections. The weighted *R*-factor *wR* and goodness of fit *S* are based on *F*^2^, conventional *R*-factors *R* are based on *F*, with *F* set to zero for negative *F*^2^. The threshold expression of *F*^2^ > σ(*F*^2^) is used only for calculating *R*-factors(gt) *etc*. and is not relevant to the choice of reflections for refinement. *R*-factors based on *F*^2^ are statistically about twice as large as those based on *F*, and *R*- factors based on ALL data will be even larger.

### Fractional atomic coordinates and isotropic or equivalent isotropic displacement parameters (Å^2^)

**Table:**

	*x*	*y*	*z*	*U*_iso_*/*U*_eq_	Occ. (<1)
Zn1	0.14673 (3)	0.98758 (4)	0.15309 (1)	0.0276 (1)	
O1	0.15029 (16)	1.0865 (2)	0.22605 (8)	0.0289 (7)	
O2	0.25273 (17)	1.0801 (2)	0.30401 (8)	0.0274 (6)	
O3	0.21577 (18)	0.8234 (2)	0.09690 (8)	0.0314 (7)	
O4	0.3476 (2)	0.6508 (3)	0.09550 (10)	0.0689 (10)	
O5	0.5868 (2)	0.7374 (2)	0.27002 (9)	0.0447 (8)	
O6	−0.00735 (17)	0.8304 (2)	0.17669 (8)	0.0297 (7)	
O7	−0.19803 (19)	0.7776 (2)	0.14763 (9)	0.0401 (8)	
O8	0.20512 (17)	1.1572 (2)	0.11038 (8)	0.0322 (7)	
O9	0.1454 (2)	1.2917 (2)	0.04327 (9)	0.0413 (8)	
O10	−0.26324 (18)	1.0555 (2)	−0.01266 (8)	0.0389 (8)	
N1	0.28365 (19)	0.8892 (2)	0.19133 (9)	0.0215 (7)	
N2	0.00293 (18)	1.0208 (2)	0.10403 (9)	0.0213 (7)	
C1	0.3153 (2)	0.9352 (3)	0.23913 (11)	0.0201 (8)	
C2	0.4150 (2)	0.8856 (3)	0.26789 (11)	0.0228 (8)	
C3	0.4854 (2)	0.7857 (3)	0.24588 (12)	0.0278 (10)	
C4	0.4493 (3)	0.7357 (3)	0.19700 (12)	0.0281 (9)	
C5	0.3486 (2)	0.7895 (3)	0.17071 (11)	0.0230 (9)	
C6	0.2327 (2)	1.0437 (3)	0.25746 (11)	0.0220 (8)	
C7	0.3012 (3)	0.7494 (3)	0.11652 (12)	0.0317 (10)	
C8	−0.0969 (2)	0.9428 (3)	0.10265 (11)	0.0222 (8)	
C9	−0.1869 (2)	0.9531 (3)	0.06417 (11)	0.0270 (9)	
C10	−0.1751 (2)	1.0492 (3)	0.02501 (11)	0.0260 (9)	
C11	−0.0730 (2)	1.1329 (3)	0.02694 (11)	0.0237 (9)	
C12	0.0138 (2)	1.1140 (3)	0.06687 (11)	0.0218 (9)	
C13	−0.0976 (2)	0.8425 (3)	0.14675 (11)	0.0255 (9)	
C14	0.1313 (3)	1.1960 (3)	0.07331 (12)	0.0279 (10)	
N3	0.8557 (2)	0.4051 (2)	0.10378 (9)	0.0258 (8)	
N4	0.6479 (2)	0.2623 (3)	0.08062 (10)	0.0367 (9)	
C15	0.9555 (2)	0.4806 (3)	0.11164 (11)	0.0291 (9)	
C16	1.0300 (3)	0.4557 (3)	0.15657 (12)	0.0356 (11)	
C17	1.0020 (3)	0.3569 (3)	0.19104 (13)	0.0336 (10)	
C18	0.8955 (3)	0.2793 (3)	0.18217 (12)	0.0280 (9)	
C19	0.8607 (3)	0.1741 (3)	0.21645 (13)	0.0350 (11)	
C20	0.7593 (3)	0.1014 (3)	0.20556 (13)	0.0383 (11)	
C21	0.6825 (3)	0.1273 (3)	0.15952 (12)	0.0333 (10)	
C22	0.5761 (3)	0.0541 (4)	0.14581 (16)	0.0508 (12)	
C23	0.5108 (3)	0.0864 (4)	0.10097 (16)	0.0546 (16)	
C24	0.5479 (3)	0.1905 (4)	0.06875 (14)	0.0463 (13)	
C25	0.7131 (3)	0.2303 (3)	0.12519 (12)	0.0301 (10)	
C26	0.8223 (3)	0.3064 (3)	0.13721 (12)	0.0267 (9)	
C27	0.9843 (3)	0.5828 (3)	0.07228 (13)	0.0380 (11)	
C28	0.4762 (3)	0.2261 (5)	0.01921 (16)	0.0668 (18)	
O1W	0.7545 (3)	0.4750 (3)	0.00253 (10)	0.0581 (10)	
O2W	0.6528 (3)	0.8705 (3)	0.35323 (11)	0.0646 (11)	
O3W	0.5323 (7)	0.4978 (8)	0.0999 (3)	0.067 (3)	0.350
H2	0.43540	0.91820	0.30170	0.0270*	
H4	0.49330	0.66560	0.18210	0.0340*	
H5	0.59810	0.77430	0.29900	0.0670*	
H7	−0.19040	0.71470	0.16920	0.0600*	
H9	−0.25570	0.89670	0.06400	0.0320*	
H10	−0.24150	1.10420	−0.03660	0.0580*	
H11	−0.06410	1.20040	0.00160	0.0280*	
H3	0.80910	0.41970	0.07570	0.0310*	
H16	1.10080	0.50780	0.16320	0.0430*	
H17	1.05410	0.34060	0.22090	0.0400*	
H19	0.90950	0.15550	0.24700	0.0420*	
H20	0.73840	0.03220	0.22850	0.0460*	
H22	0.55050	−0.01610	0.16730	0.0610*	
H23	0.43930	0.03780	0.09140	0.0660*	
H27A	0.91050	0.62890	0.05940	0.0570*	
H27B	1.02020	0.53750	0.04370	0.0570*	
H27C	1.04090	0.64920	0.08790	0.0570*	
H28A	0.44320	0.31740	0.02190	0.1000*	
H28B	0.52840	0.22280	−0.00940	0.1000*	
H28C	0.41050	0.16120	0.01300	0.1000*	
H1WA	0.774 (4)	0.546 (3)	−0.0123 (16)	0.0870*	
H1WB	0.713 (4)	0.427 (3)	−0.0189 (14)	0.0870*	
H2WA	0.687 (4)	0.814 (3)	0.3724 (16)	0.0970*	
H2WB	0.657 (3)	0.948 (2)	0.3640 (17)	0.0970*	
H3WA	0.483 (6)	0.559 (4)	0.105 (5)	0.1010*	0.350
H3WB	0.523 (3)	0.430 (3)	0.1179 (8)	0.1010*	0.350

### Atomic displacement parameters (Å^2^)

**Table:**

	*U*^11^	*U*^22^	*U*^33^	*U*^12^	*U*^13^	*U*^23^
Zn1	0.0267 (2)	0.0298 (2)	0.0248 (2)	0.0004 (2)	−0.0081 (1)	0.0028 (2)
O1	0.0257 (10)	0.0283 (12)	0.0313 (12)	0.0070 (9)	−0.0067 (9)	−0.0021 (10)
O2	0.0335 (11)	0.0264 (11)	0.0219 (11)	0.0065 (9)	−0.0003 (9)	−0.0041 (9)
O3	0.0339 (12)	0.0381 (13)	0.0211 (11)	−0.0020 (10)	−0.0047 (9)	−0.0005 (10)
O4	0.0615 (17)	0.091 (2)	0.0508 (17)	0.0338 (16)	−0.0196 (13)	−0.0456 (16)
O5	0.0410 (13)	0.0526 (16)	0.0375 (14)	0.0277 (11)	−0.0168 (11)	−0.0113 (12)
O6	0.0293 (11)	0.0335 (12)	0.0253 (11)	−0.0030 (9)	−0.0039 (9)	0.0067 (10)
O7	0.0355 (12)	0.0417 (14)	0.0419 (14)	−0.0137 (11)	−0.0056 (10)	0.0226 (12)
O8	0.0279 (11)	0.0344 (12)	0.0324 (13)	−0.0099 (9)	−0.0108 (9)	0.0035 (10)
O9	0.0481 (14)	0.0333 (13)	0.0408 (14)	−0.0172 (11)	−0.0079 (11)	0.0170 (12)
O10	0.0306 (11)	0.0603 (16)	0.0238 (12)	−0.0119 (10)	−0.0114 (9)	0.0133 (11)
N1	0.0228 (12)	0.0219 (12)	0.0195 (12)	−0.0016 (10)	−0.0009 (9)	−0.0034 (10)
N2	0.0232 (11)	0.0196 (12)	0.0208 (11)	−0.0016 (10)	−0.0006 (9)	−0.0015 (11)
C1	0.0240 (14)	0.0176 (14)	0.0187 (14)	−0.0021 (11)	0.0015 (11)	0.0000 (12)
C2	0.0258 (14)	0.0244 (15)	0.0172 (14)	0.0016 (12)	−0.0045 (11)	0.0009 (13)
C3	0.0268 (16)	0.0275 (17)	0.0281 (17)	0.0076 (13)	−0.0052 (13)	0.0011 (14)
C4	0.0331 (16)	0.0248 (16)	0.0260 (16)	0.0078 (13)	0.0005 (13)	−0.0041 (14)
C5	0.0272 (15)	0.0217 (15)	0.0197 (15)	0.0014 (12)	−0.0002 (12)	−0.0040 (13)
C6	0.0210 (13)	0.0236 (16)	0.0211 (15)	−0.0017 (11)	−0.0007 (11)	0.0020 (12)
C7	0.0308 (17)	0.039 (2)	0.0250 (17)	−0.0003 (14)	0.0009 (13)	−0.0107 (15)
C8	0.0198 (14)	0.0242 (15)	0.0224 (15)	−0.0023 (11)	0.0009 (11)	0.0015 (12)
C9	0.0218 (14)	0.0349 (18)	0.0238 (16)	−0.0066 (12)	−0.0008 (12)	0.0015 (13)
C10	0.0224 (14)	0.0356 (17)	0.0192 (15)	0.0027 (12)	−0.0047 (12)	−0.0002 (13)
C11	0.0285 (15)	0.0241 (16)	0.0179 (14)	0.0016 (12)	−0.0018 (12)	0.0036 (13)
C12	0.0263 (15)	0.0193 (15)	0.0196 (15)	−0.0010 (12)	0.0011 (11)	0.0001 (13)
C13	0.0256 (15)	0.0264 (16)	0.0242 (16)	−0.0045 (13)	−0.0004 (12)	0.0017 (14)
C14	0.0305 (16)	0.0258 (17)	0.0266 (17)	−0.0064 (13)	−0.0025 (13)	0.0003 (14)
N3	0.0271 (13)	0.0261 (14)	0.0240 (13)	−0.0001 (11)	−0.0002 (10)	−0.0024 (11)
N4	0.0306 (14)	0.0492 (18)	0.0300 (15)	−0.0070 (12)	0.0001 (12)	0.0005 (13)
C15	0.0306 (15)	0.0296 (17)	0.0273 (15)	−0.0015 (13)	0.0036 (12)	−0.0037 (15)
C16	0.0342 (17)	0.038 (2)	0.0338 (18)	−0.0056 (14)	−0.0037 (14)	−0.0048 (15)
C17	0.0322 (17)	0.0376 (19)	0.0295 (18)	0.0059 (14)	−0.0083 (13)	−0.0032 (16)
C18	0.0340 (16)	0.0254 (16)	0.0244 (16)	0.0064 (13)	0.0010 (13)	−0.0027 (14)
C19	0.0419 (19)	0.0329 (18)	0.0302 (18)	0.0104 (15)	0.0027 (14)	−0.0008 (15)
C20	0.051 (2)	0.0303 (19)	0.0353 (19)	0.0047 (16)	0.0148 (16)	0.0063 (16)
C21	0.0373 (17)	0.0335 (18)	0.0302 (18)	−0.0054 (14)	0.0091 (14)	0.0008 (15)
C22	0.048 (2)	0.052 (2)	0.054 (2)	−0.0172 (17)	0.0154 (19)	0.0017 (19)
C23	0.040 (2)	0.069 (3)	0.055 (3)	−0.0253 (19)	0.0050 (18)	0.001 (2)
C24	0.0295 (17)	0.065 (3)	0.044 (2)	−0.0114 (17)	0.0000 (15)	−0.0022 (19)
C25	0.0307 (16)	0.0339 (18)	0.0257 (16)	0.0009 (13)	0.0017 (13)	−0.0038 (14)
C26	0.0301 (16)	0.0248 (16)	0.0253 (16)	0.0024 (13)	0.0030 (13)	−0.0027 (14)
C27	0.0466 (19)	0.0337 (19)	0.0338 (19)	−0.0114 (15)	0.0041 (15)	−0.0014 (16)
C28	0.035 (2)	0.106 (4)	0.057 (3)	−0.020 (2)	−0.0126 (18)	0.006 (3)
O1W	0.0858 (19)	0.0464 (17)	0.0386 (14)	−0.0232 (14)	−0.0184 (13)	0.0125 (13)
O2W	0.081 (2)	0.0485 (17)	0.0573 (18)	0.0233 (15)	−0.0433 (15)	−0.0106 (15)
O3W	0.065 (5)	0.047 (5)	0.087 (6)	0.020 (4)	−0.011 (4)	−0.001 (5)

### Geometric parameters (Å, °)

**Table:**

Zn1—O1	2.113 (2)	C4—C5	1.367 (4)
Zn1—O3	2.329 (2)	C5—C7	1.508 (4)
Zn1—O6	2.408 (2)	C8—C13	1.503 (4)
Zn1—O8	2.119 (2)	C8—C9	1.358 (4)
Zn1—N1	1.995 (2)	C9—C10	1.393 (4)
Zn1—N2	1.987 (2)	C10—C11	1.394 (4)
O1—C6	1.247 (3)	C11—C12	1.369 (4)
O2—C6	1.256 (3)	C12—C14	1.527 (4)
O3—C7	1.267 (4)	C2—H2	0.9400
O4—C7	1.237 (4)	C4—H4	0.9400
O5—C3	1.330 (3)	C9—H9	0.9400
O6—C13	1.223 (3)	C11—H11	0.9400
O7—C13	1.282 (3)	C15—C27	1.476 (4)
O8—C14	1.270 (4)	C15—C16	1.394 (4)
O9—C14	1.233 (4)	C16—C17	1.364 (4)
O10—C10	1.327 (3)	C17—C18	1.407 (5)
O5—H5	0.8300	C18—C19	1.428 (4)
O7—H7	0.8300	C18—C26	1.391 (4)
O10—H10	0.8300	C19—C20	1.341 (5)
O1W—H1WB	0.84 (4)	C20—C21	1.431 (5)
O1W—H1WA	0.83 (3)	C21—C25	1.399 (4)
O2W—H2WB	0.81 (2)	C21—C22	1.402 (5)
O2W—H2WA	0.82 (4)	C22—C23	1.355 (6)
O3W—H3WB	0.82 (3)	C23—C24	1.395 (5)
O3W—H3WA	0.83 (6)	C24—C28	1.495 (5)
N1—C1	1.335 (4)	C25—C26	1.434 (5)
N1—C5	1.344 (3)	C16—H16	0.9400
N2—C12	1.335 (4)	C17—H17	0.9400
N2—C8	1.342 (3)	C19—H19	0.9400
N3—C26	1.363 (4)	C20—H20	0.9400
N3—C15	1.332 (3)	C22—H22	0.9400
N4—C25	1.349 (4)	C23—H23	0.9400
N4—C24	1.328 (4)	C27—H27B	0.9700
N3—H3	0.8700	C27—H27A	0.9700
C1—C6	1.500 (4)	C27—H27C	0.9700
C1—C2	1.373 (4)	C28—H28C	0.9700
C2—C3	1.396 (4)	C28—H28A	0.9700
C3—C4	1.383 (4)	C28—H28B	0.9700
			
O1—Zn1—O3	152.10 (8)	O6—C13—C8	119.5 (2)
O1—Zn1—O6	92.05 (7)	O7—C13—C8	112.9 (2)
O1—Zn1—O8	96.70 (8)	O6—C13—O7	127.6 (3)
O1—Zn1—N1	79.45 (8)	O9—C14—C12	118.5 (3)
O1—Zn1—N2	116.99 (8)	O8—C14—O9	126.6 (3)
O3—Zn1—O6	89.23 (7)	O8—C14—C12	114.9 (3)
O3—Zn1—O8	95.25 (8)	C1—C2—H2	121.00
O3—Zn1—N1	72.69 (8)	C3—C2—H2	121.00
O3—Zn1—N2	90.06 (8)	C5—C4—H4	120.00
O6—Zn1—O8	151.69 (7)	C3—C4—H4	120.00
O6—Zn1—N1	95.35 (8)	C8—C9—H9	121.00
O6—Zn1—N2	73.50 (8)	C10—C9—H9	121.00
O8—Zn1—N1	112.68 (8)	C12—C11—H11	121.00
O8—Zn1—N2	78.55 (8)	C10—C11—H11	121.00
N1—Zn1—N2	159.81 (9)	N3—C15—C27	119.3 (2)
Zn1—O1—C6	112.91 (18)	N3—C15—C16	117.6 (3)
Zn1—O3—C7	114.54 (19)	C16—C15—C27	123.1 (3)
Zn1—O6—C13	109.86 (18)	C15—C16—C17	121.1 (3)
Zn1—O8—C14	114.93 (18)	C16—C17—C18	120.3 (3)
C3—O5—H5	109.00	C17—C18—C26	117.6 (3)
C13—O7—H7	109.00	C19—C18—C26	119.1 (3)
C10—O10—H10	109.00	C17—C18—C19	123.3 (3)
H1WA—O1W—H1WB	108 (4)	C18—C19—C20	120.8 (3)
H2WA—O2W—H2WB	115 (4)	C19—C20—C21	121.1 (3)
H3WA—O3W—H3WB	113 (7)	C20—C21—C22	123.8 (3)
Zn1—N1—C5	124.33 (19)	C22—C21—C25	116.5 (3)
Zn1—N1—C1	115.63 (17)	C20—C21—C25	119.7 (3)
C1—N1—C5	119.9 (2)	C21—C22—C23	118.8 (3)
Zn1—N2—C8	122.97 (18)	C22—C23—C24	121.1 (3)
Zn1—N2—C12	117.32 (16)	N4—C24—C28	117.1 (3)
C8—N2—C12	119.2 (2)	N4—C24—C23	121.7 (3)
C15—N3—C26	123.8 (3)	C23—C24—C28	121.2 (3)
C24—N4—C25	117.3 (3)	C21—C25—C26	118.5 (3)
C26—N3—H3	118.00	N4—C25—C21	124.5 (3)
C15—N3—H3	118.00	N4—C25—C26	117.0 (3)
C2—C1—C6	124.4 (3)	C18—C26—C25	120.8 (3)
N1—C1—C6	113.9 (2)	N3—C26—C25	119.6 (3)
N1—C1—C2	121.8 (2)	N3—C26—C18	119.6 (3)
C1—C2—C3	118.6 (3)	C15—C16—H16	119.00
O5—C3—C4	118.6 (3)	C17—C16—H16	119.00
O5—C3—C2	122.4 (3)	C16—C17—H17	120.00
C2—C3—C4	119.0 (2)	C18—C17—H17	120.00
C3—C4—C5	119.1 (3)	C20—C19—H19	120.00
N1—C5—C7	113.3 (2)	C18—C19—H19	120.00
C4—C5—C7	125.1 (3)	C19—C20—H20	119.00
N1—C5—C4	121.6 (3)	C21—C20—H20	119.00
O2—C6—C1	115.6 (2)	C21—C22—H22	121.00
O1—C6—C1	117.9 (2)	C23—C22—H22	121.00
O1—C6—O2	126.5 (3)	C24—C23—H23	119.00
O3—C7—O4	126.4 (3)	C22—C23—H23	119.00
O3—C7—C5	114.9 (3)	C15—C27—H27B	109.00
O4—C7—C5	118.8 (3)	H27A—C27—H27C	109.00
N2—C8—C13	113.7 (2)	C15—C27—H27C	110.00
N2—C8—C9	122.4 (3)	H27A—C27—H27B	110.00
C9—C8—C13	123.9 (2)	C15—C27—H27A	109.00
C8—C9—C10	118.5 (2)	H27B—C27—H27C	110.00
O10—C10—C11	123.4 (3)	C24—C28—H28C	109.00
O10—C10—C9	117.3 (2)	H28A—C28—H28C	109.00
C9—C10—C11	119.3 (2)	H28B—C28—H28C	109.00
C10—C11—C12	118.1 (3)	H28A—C28—H28B	109.00
N2—C12—C11	122.5 (2)	C24—C28—H28A	109.00
C11—C12—C14	123.8 (3)	C24—C28—H28B	109.00
N2—C12—C14	113.7 (2)		
			
O3—Zn1—O1—C6	4.4 (3)	C26—N3—C15—C27	178.7 (3)
O6—Zn1—O1—C6	96.58 (18)	C15—N3—C26—C25	−179.9 (3)
O8—Zn1—O1—C6	−110.39 (18)	C24—N4—C25—C21	−0.1 (5)
N1—Zn1—O1—C6	1.51 (18)	C25—N4—C24—C23	0.3 (5)
N2—Zn1—O1—C6	169.07 (17)	C25—N4—C24—C28	−179.8 (3)
O1—Zn1—O3—C7	−0.9 (3)	C24—N4—C25—C26	178.8 (3)
O6—Zn1—O3—C7	−93.9 (2)	N1—C1—C2—C3	0.9 (4)
O8—Zn1—O3—C7	114.1 (2)	N1—C1—C6—O2	173.5 (2)
N1—Zn1—O3—C7	2.0 (2)	C6—C1—C2—C3	−179.1 (3)
N2—Zn1—O3—C7	−167.4 (2)	N1—C1—C6—O1	−5.0 (4)
O1—Zn1—O6—C13	119.77 (19)	C2—C1—C6—O1	175.0 (3)
O3—Zn1—O6—C13	−88.10 (19)	C2—C1—C6—O2	−6.6 (4)
O8—Zn1—O6—C13	11.6 (3)	C1—C2—C3—C4	−3.0 (4)
N1—Zn1—O6—C13	−160.63 (19)	C1—C2—C3—O5	176.4 (3)
N2—Zn1—O6—C13	2.19 (19)	C2—C3—C4—C5	2.7 (4)
O1—Zn1—O8—C14	−120.2 (2)	O5—C3—C4—C5	−176.7 (3)
O3—Zn1—O8—C14	85.1 (2)	C3—C4—C5—C7	177.5 (3)
O6—Zn1—O8—C14	−13.1 (3)	C3—C4—C5—N1	−0.3 (4)
N1—Zn1—O8—C14	158.5 (2)	N1—C5—C7—O3	6.4 (4)
N2—Zn1—O8—C14	−3.9 (2)	N1—C5—C7—O4	−173.9 (3)
O1—Zn1—N1—C1	−4.36 (18)	C4—C5—C7—O4	8.1 (5)
O1—Zn1—N1—C5	−179.5 (2)	C4—C5—C7—O3	−171.6 (3)
O3—Zn1—N1—C1	177.0 (2)	N2—C8—C9—C10	−0.6 (4)
O3—Zn1—N1—C5	2.0 (2)	C13—C8—C9—C10	−179.2 (3)
O6—Zn1—N1—C1	−95.45 (19)	N2—C8—C13—O6	−6.4 (4)
O6—Zn1—N1—C5	89.5 (2)	N2—C8—C13—O7	174.0 (2)
O8—Zn1—N1—C1	88.55 (19)	C9—C8—C13—O6	172.4 (3)
O8—Zn1—N1—C5	−86.5 (2)	C9—C8—C13—O7	−7.3 (4)
N2—Zn1—N1—C1	−150.6 (2)	C8—C9—C10—O10	178.8 (2)
N2—Zn1—N1—C5	34.3 (4)	C8—C9—C10—C11	−1.1 (4)
O1—Zn1—N2—C8	−90.0 (2)	C9—C10—C11—C12	1.9 (4)
O1—Zn1—N2—C12	98.8 (2)	O10—C10—C11—C12	−178.0 (3)
O3—Zn1—N2—C8	83.0 (2)	C10—C11—C12—C14	178.7 (3)
O3—Zn1—N2—C12	−88.3 (2)	C10—C11—C12—N2	−1.2 (4)
O6—Zn1—N2—C8	−6.2 (2)	N2—C12—C14—O8	5.1 (4)
O6—Zn1—N2—C12	−177.5 (2)	C11—C12—C14—O8	−174.8 (3)
O8—Zn1—N2—C8	178.3 (2)	C11—C12—C14—O9	5.7 (4)
O8—Zn1—N2—C12	7.01 (19)	N2—C12—C14—O9	−174.4 (3)
N1—Zn1—N2—C8	52.2 (4)	N3—C15—C16—C17	0.4 (4)
N1—Zn1—N2—C12	−119.1 (3)	C27—C15—C16—C17	−177.7 (3)
Zn1—O1—C6—O2	−176.9 (2)	C15—C16—C17—C18	−1.1 (5)
Zn1—O1—C6—C1	1.3 (3)	C16—C17—C18—C19	179.8 (3)
Zn1—O3—C7—O4	175.4 (3)	C16—C17—C18—C26	0.7 (5)
Zn1—O3—C7—C5	−4.9 (3)	C17—C18—C19—C20	−178.7 (3)
Zn1—O6—C13—O7	−178.8 (2)	C26—C18—C19—C20	0.4 (5)
Zn1—O6—C13—C8	1.5 (3)	C17—C18—C26—N3	0.2 (4)
Zn1—O8—C14—O9	−180.0 (3)	C17—C18—C26—C25	179.2 (3)
Zn1—O8—C14—C12	0.6 (3)	C19—C18—C26—N3	−178.9 (3)
Zn1—N1—C1—C2	−173.8 (2)	C19—C18—C26—C25	0.1 (5)
Zn1—N1—C1—C6	6.2 (3)	C18—C19—C20—C21	−0.4 (5)
C5—N1—C1—C2	1.5 (4)	C19—C20—C21—C22	179.4 (3)
C5—N1—C1—C6	−178.5 (2)	C19—C20—C21—C25	−0.2 (5)
Zn1—N1—C5—C4	173.1 (2)	C20—C21—C22—C23	−179.3 (3)
Zn1—N1—C5—C7	−5.0 (3)	C25—C21—C22—C23	0.2 (5)
C1—N1—C5—C4	−1.8 (4)	C20—C21—C25—N4	179.4 (3)
C1—N1—C5—C7	−179.9 (2)	C20—C21—C25—C26	0.6 (4)
Zn1—N2—C8—C9	−169.8 (2)	C22—C21—C25—N4	−0.2 (5)
Zn1—N2—C8—C13	9.0 (3)	C22—C21—C25—C26	−179.0 (3)
C12—N2—C8—C9	1.3 (4)	C21—C22—C23—C24	0.0 (6)
C12—N2—C8—C13	−179.9 (2)	C22—C23—C24—N4	−0.2 (6)
Zn1—N2—C12—C11	171.2 (2)	C22—C23—C24—C28	179.9 (4)
Zn1—N2—C12—C14	−8.7 (3)	N4—C25—C26—N3	−0.5 (4)
C8—N2—C12—C11	−0.4 (4)	N4—C25—C26—C18	−179.5 (3)
C8—N2—C12—C14	179.7 (2)	C21—C25—C26—N3	178.4 (3)
C15—N3—C26—C18	−0.8 (4)	C21—C25—C26—C18	−0.6 (5)
C26—N3—C15—C16	0.5 (4)		

### Hydrogen-bond geometry (Å, °)

**Table:**

Cg1 is the centroid of the N1,C1–C5 ring.

**Table:**

*D*—H···*A*	*D*—H	H···*A*	*D*···*A*	*D*—H···*A*
N3—H3···N4	0.87	2.37	2.721 (3)	105
N3—H3···O1W	0.87	2.01	2.848 (4)	161
O5—H5···O2W	0.83	1.76	2.570 (4)	166
O7—H7···O2^i^	0.83	1.66	2.402 (3)	147
O10—H10···O3^ii^	0.83	1.75	2.562 (3)	166
O1W—H1WA···O9^iii^	0.83 (3)	2.02 (4)	2.833 (4)	169 (4)
O1W—H1WB···O4^iv^	0.84 (4)	2.18 (4)	2.960 (4)	156 (3)
O2W—H2WA···O8^v^	0.82 (4)	1.98 (4)	2.738 (4)	155 (4)
O2W—H2WB···O4^vi^	0.81 (2)	2.25 (3)	3.046 (4)	171 (4)
O3W—H3WA···O4	0.83 (2)	1.75 (5)	2.530 (7)	157 (13)
O3W—H3WB···O2W^v^	0.82 (2)	2.21 (4)	2.750 (9)	123 (3)
C27—H27C···O3^vii^	0.97	2.58	3.504 (4)	160
C22—H22···Cg1^viii^	0.94	2.76	3.634 (4)	155

Symmetry codes: (i) −*x*, *y*−1/2, −*z*+1/2; (ii) −*x*, −*y*+2, −*z*; (iii) −*x*+1, −*y*+2, −*z*; (iv) −*x*+1, −*y*+1, −*z*; (v) −*x*+1, *y*−1/2, −*z*+1/2; (vi) −*x*+1, *y*+1/2, −*z*+1/2; (vii) *x*+1, *y*, *z*; (viii) *x*, *y*−1, *z*.
